# Prospective Application of Tannic Acid in Acetaminophen (APAP)-Induced Acute Liver Failure

**DOI:** 10.3390/ijms25010317

**Published:** 2023-12-25

**Authors:** Yong-Heng Lin, Yu-Che Lin, Yung-Te Hou

**Affiliations:** Department of Biomechatronics Engineering, National Taiwan University, No. 1, Sec. 4, Roosevelt Road, Taipei 10617, Taiwan; r11631030@ntu.edu.tw (Y.-H.L.); r08631039@ntu.edu.tw (Y.-C.L.)

**Keywords:** liver regeneration, tannic acid, hepatoprotective and hepatocurative, acetaminophen-induced liver injury

## Abstract

This study investigated the effect of tannic acid (TA), a natural plant-derived polyphenol, on hepatocyte viability and function, focusing on both hepatoprotective and hepatocurative aspects within liver failure models. In an in vitro prevention model, the TA-containing group exhibited 1.5-fold and 59-fold higher relative cell viability and albumin synthesis, respectively, in injured mature hepatocytes (MHs) and 1.14-fold and 1.10-fold higher values in injured small hepatocytes (SHs), compared with the TA-free group. In the in vitro curative model, the TA-containing group exhibited 3.25-fold and 113-fold higher relative cell viability and albumin synthesis, respectively, in injured MHs and 0.36-fold and 3.55-fold higher values in injured SHs, compared with the TA-free group. In the in vivo disease model, the administration of 300 μL of 1 μg/mL TA significantly mitigated acute liver failure damage and post-APAP toxicity in mice. This was evident in serum analysis, where the levels of alanine transaminase, aspartate aminotransferase, and total bilirubin notably decreased, in agreement with histological observations. The study findings reveal that TA can enhance hepatic function at specific additive concentrations. Furthermore, even when injured by APAP, hepatocytes could revert to their preinjury state after additional TA supplementation. Additionally, pretreating hepatocytes with TA can alleviate subsequent damage. Thus, TA holds clinical potential in the treatment of APAP-induced liver failure.

## 1. Introduction

Liver failure, which can be broadly categorized into acute and chronic hepatic failure, is associated with high mortality rates. Although acute liver failure is rare, it is a severe syndrome with a high risk of mortality [1]. In developed countries, drug-related problems, especially acetaminophen (APAP) misuse and abuse, are a major cause of acute liver failure [2]. Under normal conditions, the metabolism of APAP generates free radicals, which are typically neutralized by the body’s antioxidant defense system. However, when this balance is disrupted, these radicals cause oxidative stress, leading to liver cell injury [3,4]. The mechanism of APAP toxicity involves its liver metabolism, producing the toxic n-acetyl-p-benzoquinone imine (NAPQI) metabolite. NAPQI, a key driver of APAP toxicity, swiftly reacts with and depletes glutathione (GSH). In overdose scenarios, depleted GSH enables excess NAPQI to irreversibly bind to cellular proteins, including mitochondria. This accumulation induces hepatocellular damage by escalating oxidative stress alongside mitochondrial dysfunction [3,5]. Although APAP is safe when used within recommended doses for treating pain and fever, overdosing can lead to severe liver damage and even death [6].

Oxidative stress in APAP-induced hepatotoxicity is characterized by lipid peroxidation, mitochondrial damage, and ATP depletion [3,7]. Substances with antioxidant properties may protect against such hepatocellular injury [3]. N-acetylcysteine (NAC) is currently the only FDA-approved drug for the treatment of APAP-induced liver injury [8,9]. It has garnered significant attention as a therapeutic agent against drug-induced liver injury (DILI) due to its robust antioxidant properties, particularly its ability to augment endogenous GSH levels to counteract oxidative stress [10]. However, its clinical use is substantially restricted because of the narrow therapeutic window for acute liver injury and associated side effects [8,11]. By contrast, natural products are valuable sources of novel medicines and therapies. Compounds such as triterpenoid saponins, schisandra lignans, polysaccharides, iridoids, flavonoids, and quinones have demonstrated protective effects against APAP-induced hepatotoxicity [8]. However, improving the therapeutic window of natural hepatoprotective agents and reducing their toxicity are challenges that need to be addressed [8]. Moreover, researchers and clinicians are increasingly interested in discovering naturally sourced hepatocurative and hepatoprotective agents that can enhance treatment efficacy [12].

Tannic acid (TA), which is primarily found in various plants used as food and feed, such as food grains, fruits, wine, and tea [13,14], is a natural polyphenol with diverse therapeutic properties, including antioxidant, antitumor, and anti-inflammatory effects [15]. The hepatoprotective effects of TA on APAP-induced hepatotoxicity were previously investigated due to its anti-oxidation, anti-inflammation, and anti-apoptosis properties. Their study further demonstrated that TA treatment could alleviate hepatotoxicity in APAP-induced mice, ameliorating hepatic dysfunction and mitigating histopathological changes [3]. Although TA shows promise in anticancer applications, its mechanisms for enhancing hepatic functions in vivo remain unclear [13]. Furthermore, the dual hepatoprotective and hepatocurative effects of TA remain relatively unexplored.

Numerous studies have highlighted similarities between carbon tetrachloride (CCl_4_)-induced liver damage and human liver cirrhosis [16,17]. Therefore, CCl_4_-induced liver damage is commonly used as an experimental model for screening hepatoprotective and hepatocurative herbal medicines or drugs [16]. Although APAP and CCl_4_ are both commonly used in intrinsic drug-induced liver injury models, APAP-induced liver injury is the most clinically relevant [18]. However, few studies have focused on hepatoprotective and hepatocurative drugs specifically for APAP-induced liver injury [19]. In addition, most studies have examined the potential of herbal medicines in addressing toxin-induced liver injuries, such as those affecting alanine transaminase (ALT), alkaline phosphatase (ALP), aspartate transaminase (AST), and lactate dehydrogenase levels in serum and histological studies [16,19]. Numerous phytoconstituents have been demonstrated to protect against liver diseases in both in vitro and in vivo settings [20]. However, few of these studies have examined specific components within herbal medicine or their role in facilitating the recovery of liver function, including albumin, following toxin-induced liver injury [13].

**Figure 1:**
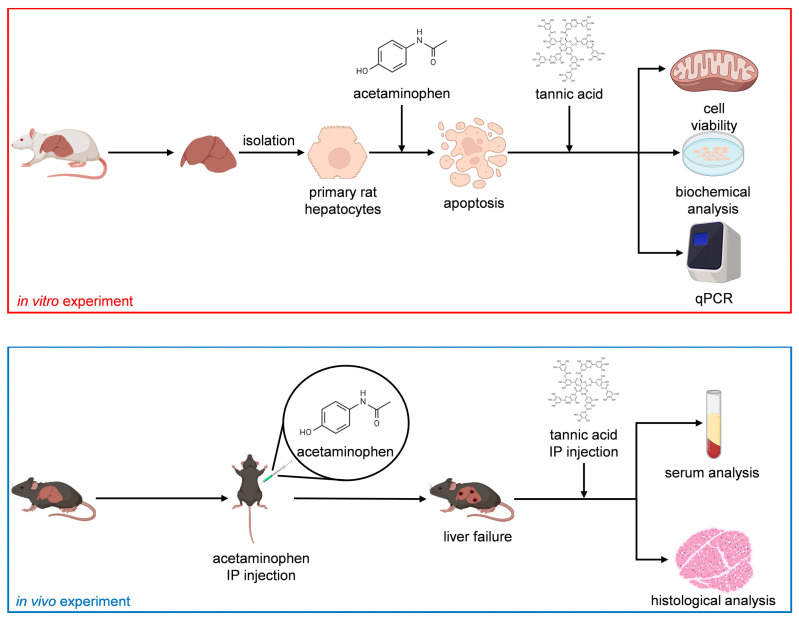
Study flowchart.

In this study, we investigated the effect of TA on the functions of normal hepatocytes. Subsequently, we established an APAP-induced acute liver failure model to determine the effects of TA on the viability and functions of injured hepatocytes, both in vitro and in vivo. In the APAP-induced acute liver failure model, TA was employed as a hepatoprotective agent prior to the introduction of APAP as a toxin and as a hepatocurative compound after APAP exposure. In addition, to assess short-term effects, we utilized primary mature hepatocytes (MHs), the gold standard in vitro cell model, because they can retain their functionality over short durations [21]. To evaluate long-term effects, we used small hepatocytes (SHs), known for their remarkable ability to proliferate and high potential to differentiate into MHs [22]. This study investigated the hepatoprotective and hepatocurative effects of TA through comprehensive in vitro and in vivo analyses. Figure 1 presents the flowchart of the study.

## 2. Results

### 2.1. Effect of TA on MHs in In Vitro Cultures

**Figure 2:**
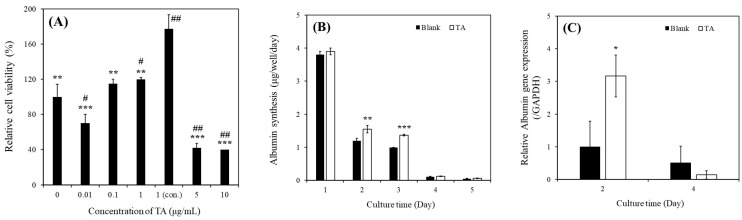

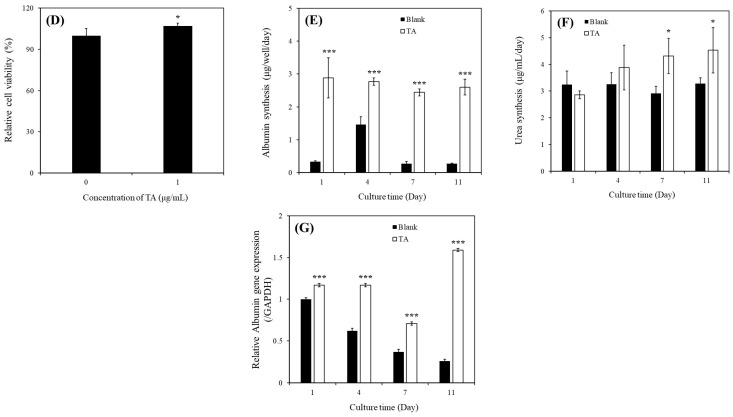
(**A**) Relative cell viability of MHs cultured in different TA conditions after 3 days of culture (** *p* < 0.01, *** *p* < 0.001, ^#^ *p <* 0.05, ^##^ *p <* 0.01, significant differences vs. continuously 1 μg/mL TA-containing group and TA-free group, respectively). (**B**) Albumin synthesis of MHs cultured in different conditions (** *p* < 0.01, *** *p* < 0.001, significant differences vs. blank on days 2 and 3, respectively). (**C**) Relative *ALB* gene expression of MHs cultured in different conditions (* *p* < 0.05, significant differences vs. blank on day 2). (**D**) Relative viability of SHs cultured in different conditions after 7 days of culture (* *p* < 0.05, significant differences vs. the TA-free group). (**E**) Albumin synthesis of SHs cultured in different conditions (*** *p* < 0.001, significant differences vs. blank on days 1, 4, 7, and 11, respectively). (**F**) Urea synthesis of SHs cultured in different conditions (* *p* < 0.05, significant differences vs. blank on days 7 and 11, respectively). (**G**) Relative *ALB* gene expression of SHs cultured in different conditions (*** *p* < 0.001, significant differences vs. blank on days 1, 4, 7, and 11, respectively). Blank: normal MHs/SHs cultured in regular Petri dishes without TA supplementation.

As depicted in Figure 2A, the relative viability of MHs cultured with 1 μg/mL of TA (120% ± 2%) was higher than that of MHs cultured in a TA-free medium (100% ± 14.35%). In addition, the relative viability of MHs cultured with continuous 1 μg/mL of TA (177.49% ± 15.79%) was higher than that of MHs cultured in the other conditions on day 3. These results indicated that the continuous supplementation of 1 μg/mL TA enhanced the viability of MHs. However, higher concentrations (>5 μg/mL) of TA can harm MHs, whereas lower concentrations (<0.1 μg/mL) might not yield positive effects. These findings are consistent with those of our previous study conducted in microfluidic system cultures [13]. Thus, this condition was subsequently used for further experiments.

As depicted in Figure 2B, albumin synthesis was significantly higher in MHs cultured with 1 μg/mL of TA than in those cultured in a TA-free medium within the first 3 days of culture. Specifically, on day 2, the albumin concentration was 1.20 ± 0.07 µg/well/day in the TA-free group and 1.55 ± 0.11 µg/well/day in the TA-containing group. On day 3, the albumin concentration was 0.99 ± 0.01 µg/well/day in the TA-free group and 1.37 ± 0.03 µg/well/day in the TA-containing group. However, the albumin synthesis in MHs markedly decreased on days 4 and 5, even in the TA-containing group. As presented in Figure 2C, the albumin (*ALB*) gene expression was significantly higher in MHs cultured with 1 μg/mL of TA than in those cultured in the TA-free condition within 2 days of culture. Specifically, on day 2, the values were 1.00 ± 0.78-fold for the TA-free group and 3.17 ± 0.64 µg/well/day for the TA-containing group. However, no significant differences were observed on day 4 between these groups. Although TA has demonstrated benefits for MH culture, the occurrence of epithelial-to-mesenchymal transition (EMT) within 5 days of culturing can hinder hepatocyte growth and function for extended periods, even under favorable conditions [15]. In addition, Chiu et al. reported that the effect of EMT was more significant within the 5 day culture period compared with that of TA [14], which is consistent with our findings (Figure 2B,C).

### 2.2. Effect of TA on SHs in In Vitro Cultures

As presented in Figure 2D, the relative viability of SHs cultured with 1 μg/mL of TA (107% ± 2.1%) was higher than that of SHs cultured in the TA-free condition (100% ± 5.2%) on day 7; this observation is consistent with that in MHs (Figure 2A).

As illustrated in Figure 2E, albumin synthesis in SHs cultured with 1 μg/mL of TA was significantly higher than that in SHs cultured in the TA-free condition within 11 days of culture. Specifically, on day 1, the values were 0.33 ± 0.03 µg/well/day for the TA-free group and 2.88 ± 0.61 µg/well/day for the TA-containing group. On day 4, the values were 1.46 ± 0.24 µg/well/day for the TA-free group and 2.77 ± 0.11 µg/well/day for the TA-containing group. On day 7, the values were 0.27 ± 0.07 µg/well/day for the TA-free group and 2.44 ± 0.11 µg/well/day for the TA-containing group. On day 11, the values were 0.27 ± 0.02 µg/well/day for the TA-free group and 2.60 ± 0.24 µg/well/day for the TA-containing group. As depicted in Figure 2F, urea synthesis in SHs cultured with 1 μg/mL of TA was significantly higher than that in SHs cultured in the TA-free condition within 11 days of culture. Specifically, on day 7, the values were 2.92 ± 0.26 µg/well/day for the TA-free group and 4.31 ± 0.66 µg/well/day for the TA-containing group. On day 11, the values were 3.28 ± 0.21 µg/well/day for the TA-free group and 4.53 ± 0.85 µg/well/day for the TA-containing group.

As presented in Figure 2G, the *ALB* gene expression in SHs cultured with 1 μg/mL of TA was significantly higher than that in SHs cultured in the TA-free condition within 11 days of culture. Specifically, on day 1, the values were 1.00 ± 0.02-fold for the TA-free group and 1.17 ± 0.02-fold for the TA-containing group. On day 4, the values were 0.62 ± 0.03-fold for the TA-free group and 1.17 ± 0.02-fold for the TA-containing group. On day 7, the values were 0.37 ± 0.03-fold for the TA-free group and 0.71 ± 0.02-fold for the TA-containing group. On day 11, the values were 0.26 ± 0.02-fold for the TA-free group and 1.59 ± 0.02-fold for the TA-containing group. These findings demonstrated that TA exerted positive effects on hepatocyte culture, enhancing both cell viability and function. Furthermore, our finding of the ability of SHs to sustain their functions over an extended period aligns with that of a previous study [13].

### 2.3. Hepatoprotective and Hepatocurative Effects of TA on APAP-Injured MHs

**Figure 3:**
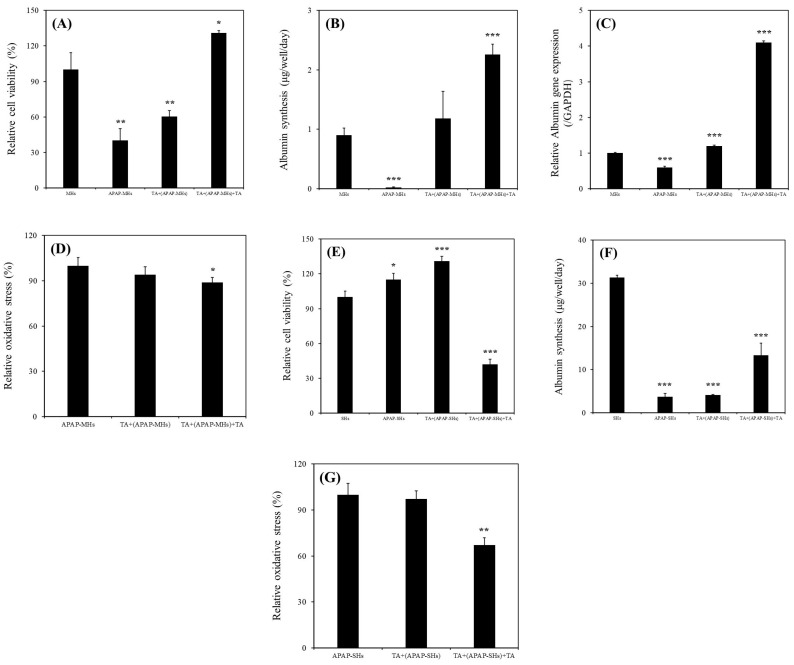
(**A**) Relative viability of MHs cultured in different conditions (* *p* < 0.05, ** *p* < 0.01, significant differences vs. the MHs group). (**B**) Albumin synthesis of MHs cultured in different conditions (*** *p* < 0.001, significant differences vs. the MHs group). (**C**) Relative *ALB* gene expression of MHs cultured in different conditions (*** *p* < 0.001, significant differences vs. the MHs group). (**D**) Relative oxidative stress of MHs cultured in different conditions (* *p* < 0.05, significant differences vs. the APAP-MHs group). MHs refer to normal MHs cultured in regular Petri dishes without TA supplementation. APAP-MHs refer to APAP-injured MHs cultured in regular Petri dishes without TA supplementation. [TA + (APAP-MHs)] refers to MHs cultured in regular Petri dishes with TA supplementation, followed by exposure to APAP-induced toxicity (prevention model). [TA + (APAP-MH) + TA] refers to MHs cultured in regular Petri dishes with TA supplementation, followed by exposure to APAP-induced toxicity for 18 h, and then recovered through additional TA supplementation for another 18 h (curative model). (**E**) Relative viability of SHs cultured in different conditions (* *p* < 0.05, *** *p* < 0.001, significant differences vs. the SHs group). (**F**) Albumin synthesis of SHs cultured in different conditions (*** *p* < 0.001, significant differences vs. the SHs group). (**G**) Relative oxidative stress of SHs cultured in different conditions (** *p* < 0.01, significant differences vs. the APAP-SHs group). SHs refer to normal SHs cultured in regular Petri dishes without TA supplementation. APAP-SHs refer to APAP-injured SHs cultured in regular Petri dishes without TA supplementation. [TA + (APAP-SHs)] refers to SHs cultured in regular Petri dishes with TA supplementation, followed by exposure to APAP-induced toxicity (prevention model). [TA + (APAP-SHs) + TA] refers to SHs cultured in regular Petri dishes with TA supplementation, followed by exposure to APAP-induced toxicity for 48 h, and then recovered through additional TA supplementation for another 24 h (curative model).

As presented in Figure 3A, the relative viability of MHs cultured in the MHs, APAP-MHs, [TA + (APAP-MHs)], and [TA + (APAP-MHs) + TA] groups were 100% ± 14.35%, 40.23% ± 10.12%, 60.51% ± 8.37%, and 130.87 ± 6.25%, respectively. As illustrated in Figure 3B, albumin synthesis values in MHs cultured in the MHs, APAP-MHs, [TA + (APAP-MHs)], and [TA + (APAP-MHs) + TA] groups were 0.9 ± 0.12, 0.02 ± 0.01, 1.18 ± 0.46, and 2.26 ± 0.17 µg/well/day, respectively. As depicted in Figure 3C, *ALB* gene expression levels in MHs cultured in the MHs, APAP-MHs, [TA + (APAP-MHs)], and [TA + (APAP-MHs) + TA] groups were 1.0 ± 0.02-fold, 0.6 ± 0.03-fold, 1.2 ± 0.02-fold, and 4.1 ± 0.05-fold, respectively. These findings demonstrated that the addition of TA not only mitigated the cytotoxicity of injured hepatocytes but also enhanced their functionality. In addition, in terms of prevention, TA supplementation preconditions the functions of hepatocytes, enabling them to effectively counteract the toxic effects of APAP.

The relative oxidation stress levels in MHs cultured in the APAP-MHs, [TA + (APAP-MHs)], and [TA + (APAP-MHs) + TA] groups were 100% ± 5.4%, 94% ± 5.3%, and 89% ± 3.2%, respectively (Figure 3D), suggesting that 1 μg/mL of TA not only slightly prevents oxidative stress before toxicity but also reduces oxidative stress after cell toxicity significantly. This finding aligns with that of a previous study [23] that reported the antioxidative effects of TA, indicating that it reduces oxidative stress and free radical damage.

### 2.4. Hepatoprotective and Hepatocurative Effects of TA on APAP-Injured SHs

The relative viability values of SHs cultured in the SHs, APAP-SHs, [TA + (APAP-SHs)], and [TA + (APAP-SHs) + TA] groups were 100% ± 5.2%, 115% ± 5.3%, 131% ± 4.2%, and 42% ± 4.6%, respectively (Figure 3E). The albumin synthesis values in SHs cultured in the SHs, APAP-SHs, [TA + (APAP-SHs)], and [TA + (APAP-SHs) + TA] groups were 31.3 ± 0.55, 3.74 ± 0.71, 4.10 ± 0.17, and 13.29 ± 2.81 µg/well/day, respectively (Figure 3F). The relative oxidation stress levels in SHs cultured in the APAP-SHs, [TA + (APAP-SHs)], and [TA + (APAP-SHs) + TA] groups were 100% ± 7.4%, 97.3% ± 5.1%, and 67.1% ± 4.8%, respectively (Figure 3G), suggesting that 1 μg/mL of TA not only slightly prevents oxidative stress before toxicity but also reduces oxidative stress after cell toxicity significantly. This finding is consistent with that presented in Figure 3D.

### 2.5. Cell Apoptosis Effects of TA on APAP-Injured MHs and APAP-Injured SHs

**Figure 4:**
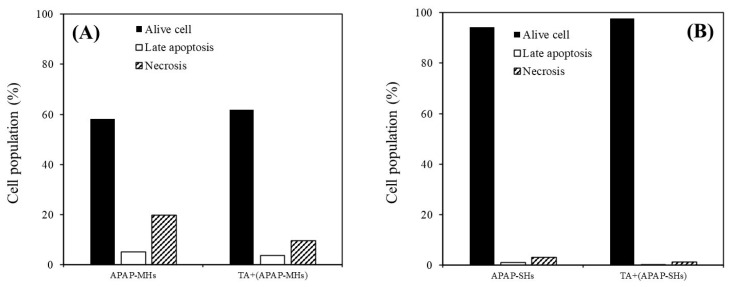
Cell apoptosis analysis was performed through flow cytometry. (**A**) The cell population of MHs cultured in different conditions. APAP-MHs refer to APAP-injured MHs cultured in regular Petri dishes without TA supplementation. [TA + (APAP-MHs)] refers to MHs cultured in regular Petri dishes with TA supplementation, followed by exposure to APAP-induced toxicity (prevention model). (**B**) The cell population of SHs cultured in different conditions. APAP-SHs refer to APAP-injured SHs cultured in regular Petri dishes without TA supplementation. [TA + (APAP-SHs)] refers to SHs cultured in regular Petri dishes with TA supplementation, followed by exposure to APAP-induced toxicity (prevention model).

As illustrated in Figure 4A, the proportions of live cells, cells in late apoptosis, and necrotic cells in MHs within the APAP-injured group were 58.1%, 5.2%, and 19.8%, respectively. In the APAP-injured group pretreated with TA, these proportions were 61.9%, 3.7%, and 9.5%, respectively. As presented in Figure 4B, the proportions of live cells, cells in late apoptosis, and necrotic cells in SHs within the APAP-injured group were 94.3%, 1.0%, and 3.0%, respectively. In the APAP-injured group pretreated with TA, these proportions were 97.7%, 0.1%, and 1.3%, respectively, consistent with the results shown in Figure 4A. Our results suggest that TA effectively prevented apoptosis in both MHs and SHs in the APAP-injured model.

### 2.6. Effect of TA on the Prevention and Recovery of APAP-Induced Liver Injury In Vivo

**Figure 5:**
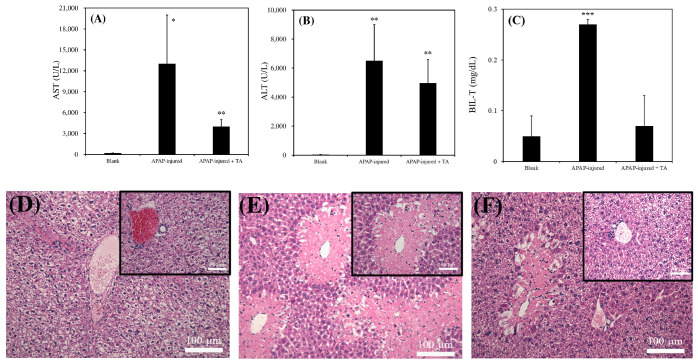
Biochemical analysis of blank, APAP-injured liver, and APAP-injured liver with TA supplementation for recovery. (**A**) AST. (**B**) ALT. (**C**) BIL-T (* *p* < 0.05, ** *p* < 0.01, *** *p* < 0.001, significant differences vs. blank). For the mouse acute liver model, 300 mg/kg of APAP was injected for poisoning, and 300 μL of 1 μg/mL TA was injected for recovery. Blank: healthy liver. Histological analysis of blank, APAP-injured liver, and APAP-injured liver with TA supplementation for recovery. (**D**) healthy liver. (**E**) APAP-injured liver. (**F**) APAP-injured liver with TA supplementation for recovery. Bars = 50 μm and 100 μm, separately.

As presented in Figure 5A, the AST level in normal mice was approximately 221 ± 35 (U/L), which is consistent with that reported in a previous study [24]. However, the AST level in 300 mg/kg APAP-induced injured mice reached 12,018 ± 6246 (U/L) after 12 h poisoning. The AST level in 300 mg/kg APAP-induced injured mice treated with 300 μL of 1 μg/mL TA was below 4005 ± 1047 (U/L). As illustrated in Figure 5B, the ALT level in normal mice was 42 ± 11 (U/L), which is consistent with that reported in a previous study [24]. The AST level in 300 mg/kg APAP-induced injured mice reached 6533 ± 2509 (U/L) after 12 h poisoning. However, the AST level in 300 mg/kg APAP-induced injured mice treated with 300 μL of 1 μg/mL TA decreased to 4971 ± 1623 (U/L). As presented in Figure 5C, the total bilirubin level (BIL-T) in normal mice was approximately 0.05 ± 0.04 (mg/dL), which is consistent with that reported in a previous study [25]. However, the BIL-T level in 300 mg/kg APAP-induced injured mice reached 0.27 ± 0.01 (mg/dL) after 12 h poisoning. The BIL-T level in 300 mg/kg APAP-induced injured mice treated with 300 μL of 1 μg/mL TA was below 0.07 ± 0.06 (mg/dL). These results revealed that mice with APAP-induced liver injury exhibited significantly higher levels of AST, ALT, and BIL-T than did normal mice. However, when mice were treated with TA after APAP poisoning, the levels of these indicators were reduced compared with those in untreated mice, indicating a potential protective effect of TA against liver damage.

Figure 5D–F presents the results of tissue slices for each group. Figure 5D illustrates clear boundaries between hepatocytes in normal mice, with fully visible veins and bile ducts. In mice treated with 300 mg/kg of APAP for 12 h (Figure 5E), numerous light pink necrotic areas were observed. The boundaries of the nuclei were blurred, and the necrotic regions were close to the veins. The histology of the damaged liver was similar to that reported in a previous study [26]. As shown in Figure 5F, the degree of necrosis in mice treated with 300 mg/kg of APAP, followed by an additional 300 mg/kg of TA, was reduced. The necrotic area in APAP-treated mice subjected to TA treatment was smaller than that in the group without TA treatment. Although we noted pink necrotic tissue near the vein, the remaining liver cells appeared relatively intact. These observations suggest that TA might alleviate APAP-induced liver damage.

## 3. Discussion

Medicinal plants have long been used for the treatment of liver diseases. Numerous studies focusing on plants and their herbal formulations worldwide have reported their hepatoprotective properties. Various phytoconstituents derived from these plants have demonstrated their effectiveness in protecting liver health, both in vitro and in vivo [20]. For instance, TA, which is abundantly found in cereals, legumes, fruits, herbs, green tea, and red wine, possesses robust antioxidant, astringent, antiviral, and antibacterial properties. TA was reported to reduce serum cholesterol and triglyceride levels and inhibit lipogenesis [27]. Moreover, these properties of TA have been reported to positively affect liver function markers, including AST, ALT, ALP, γGT, and bilirubin, in a liver injury model [13,28,29]. These properties can explain traditional beliefs in Asia that drinking tea can protect the liver. However, the term “hepatoprotective” may imply either protecting or preventing liver damage [20]. Moreover, numerous studies have explored differences between hepatoprotective and hepatocurative properties in medicinal plants [11,16]. Thus, despite the aforementioned belief, substantial empirical evidence supporting both the hepatoprotective and hepatocurative abilities of the main tea ingredient, polyphenol TA, is necessary.

APAP, which is commonly prescribed to alleviate fever and pain, is generally safe when administered in therapeutic doses. However, an overdose of APAP can lead to hepatocyte necrosis, damage to mitochondrial membranes, and an increase in mitochondrial oxidative stress [3,30]. TA exhibited substantial hepatoprotective effects against APAP-induced hepatotoxicity, indicating that the hepatoprotective mechanism of TA may be related to antioxidation, anti-inflammation, and antiapoptosis [3]. However, only a few studies have analyzed the effects of TA on albumin and urea levels in a normal liver model [13]. In addition, no study has investigated the relationship between TA and hepatic functions by using an injured liver model. To address this knowledge gap, we examined the effect of and mechanism through which TA enhances hepatocyte viability and functions in normal liver models. In addition, we investigated the hepatoprotective and hepatocurative effects of TA on APAP-induced liver failure models.

Our results revealed a significant increase in the relative viability of MHs in the presence of 1 μg/mL of TA (Figure 2A). Specifically, the continuous supplementation of 1 μg/mL of TA resulted in superior performance compared with all other conditions. Furthermore, we observed a notable enhancement in albumin synthesis in these MHs (Figure 2B). This improvement was corroborated by the upregulation of *ALB* gene expression (Figure 2C). We investigated the effects of 1 μg/mL of TA on both prevention and recovery in APAP-MHs. In the prevention model, the relative cell viability, albumin synthesis, and *ALB* gene expression in MHs were 1.5-fold, 59-fold, and 2-fold higher, respectively, in the [TA + (APAP-MHs)] group than in the APAP-MHs group. In the curative model, the relative cell viability, albumin synthesis, and *ALB* gene expression in MHs were 3.25-fold, 113-fold, and 6.83-fold higher, respectively, in the [TA + (APAP-MHs) + TA] group than in the APAP-MHs group (Figure 3A–C). These results indicated that 1 μg/mL of TA not only increased cell viability after injury but also enhanced hepatic functions, both preventing and curing APAP-induced injury. In addition, APAP damages MHs by generating free radicals. The antioxidative effect of TA exhibited a significant difference in its ability to promote recovery after toxicity, but it did not exert significant preventive effects before the onset of toxicity (Figure 3D). These results further explain the superior performance of 1 μg/mL of TA in aiding the recovery of injured MHs in a curative manner compared with its performance in preventive damage before toxicity (Figure 3A–C).

As presented in Figure 2D–G, we observed significant increases in relative viability, albumin synthesis, urea synthesis, and *ALB* gene expression in SHs in the presence of 1 μg/mL of TA within 11 days of culture. We also investigated the effects of 1 μg/mL of TA on both prevention and recovery in APAP-SHs. In the prevention model, the relative cell viability and albumin synthesis in SHs were 1.14-fold and 1.10-fold higher, respectively, in the [TA + (APAP-SHs)] group than in the APAP-SHs group. In the curative model, the relative cell viability and albumin synthesis were 0.36-fold and 3.55-fold higher, respectively, in the [TA + (APAP-SHs) + TA] group than in the APAP-SHs group (Figure 3E,F). The MTT assay does not only reflect cell viability but also cell proliferation [15,31]. Previous studies have indicated an inverse relationship between cell proliferation and differentiation [15,32]. In addition, SHs exhibited a superior ability to maintain 3D structures and resist differentiation into fibroblast-like cells compared with MHs [22]. Therefore, we hypothesize that the stimulation of APAP-SHs following TA supplementation led to advanced differentiation, resulting in the formation of more functionally mature liver tissue during in vitro proliferation (Figure 3F). The functionalized and differentiated liver tissue exhibited decreased proliferative capacity, leading to a marked decline in APAP-SHs activity (Figure 3E). Similar results were observed in normal hepatocyte cultures. The relative viability of MHs and SHs increased by 1.77-fold (day 1) and only 1.07-fold (day 7), respectively, in the TA-containing group (Figure 2A,D). However, the albumin synthesis in MHs and SHs increased by 1.38-fold (day 3) and almost 9.63-fold (day 7), respectively, in the TA-containing group (Figure 2B,E). These findings align with observations reported in prior studies [15,33].

The recovery group exhibited a significant reduction in relative oxidative stress following toxicity. The effectiveness of 1 μg/mL of TA in reducing relative oxidative stress was more pronounced in SHs than in MHs. This is because stem cells typically have lower levels of reactive oxygen species (ROS) than fully differentiated cells. Highly proliferative stem cells, such as cancer stem cells, maintain low ROS levels to prevent ROS-induced apoptosis, resulting in less DNA damage from radiation due to the presence of enhanced ROS scavenging systems [34]. However, we noted no significant preventive effects before toxicity on both MHs and SHs (Figure 3D,G). This is because the metabolic half-life of albumin in serum is approximately 17 days, and the albumin level in serum does not change rapidly [15]. Thus, with TA’s cumulative enhancement of albumin synthesis over prolonged culture periods, the disparity in albumin synthesis became increasingly evident. This difference led to a more pronounced change in the recovery of relative oxidative stress after toxicity (Figure 3G). However, although the preventive effect of TA on ROS reduction may not have been apparent, the findings shown in Figure 4 still suggest that TA effectively prevented apoptosis in both MHs and SHs in the APAP-injured model. In addition, the effectiveness of TA in ameliorating injured cell viability and inhibiting apoptosis/necrosis was more pronounced in SHs than in MHs.

We hypothesize that the mechanism underlying the effect of TA on hepatocytes under this condition may operate through alternative pathways, particularly by significantly affecting albumin synthesis. Moreover, the decrease in relative oxidative stress after toxicity might result from various factors. Apart from TA’s inherent antioxidative properties that significantly reduce free radicals after toxicity, Doweiko et al. indicated that albumin itself acts as an excellent antioxidant [35], providing protection against free radicals in hepatocytes. Xie et al. also suggested that the addition of TA strengthens protein structures [36]. Because oxidized albumin can increase oxidative stress [37], the decrease in oxidative stress can have an additive effect. This mechanism might involve the stimulation of hepatocytes by TA, leading to increased albumin synthesis [13]. In addition, the stable structure formed between TA and albumin [36] might contribute to the reduction in relative oxidative stress in the culture medium after toxicity.

In in vivo experiments, the addition of 1 μg/mL of TA in mice significantly reduced acute liver failure damage after APAP toxicity, as reflected by notable reductions in the serum levels of ALT, AST, and BIL-T (Figure 5A–C). Moreover, the histopathological examination of tissue sections revealed a significant reduction in the tissue area in the group treated with 1 μg/mL of TA compared with the untreated group (Figure 5D–F). These results demonstrated the hepatocurative effects of TA after toxicity in mice experiencing acute liver failure. These findings propose a multifaceted interaction among TA, albumin, and relative oxidative stress in hepatocytes after toxic exposure, shedding light on potential therapeutic pathways for acute liver failure. In addition, many studies have explored the therapeutic potential of TA orally in injured liver models [3,38], but only one report has discussed the potential of TA-embedded decellularized liver matrix (DLM)-based nanomedicine in promoting liver regeneration after 70% of partial hepatectomy via the intraperitoneal (IP) route [15]. However, the specific effect of TA alone via IP administration remains uncertain.

While our findings demonstrated the potential therapeutic effects of TA in an injured rat model, it is important to note that Goel et al. have reported a gradual and progressive destruction of liver cells and transformation of their architecture in *Clarias batrachus* following the IP administration of an aqueous solution of TA [39]. In addition, despite the faster and more complete absorption seen with IP administration of pharmacological agents compared to oral, intramuscular, and subcutaneous routes, it has limitations. One such limitation is first-pass metabolism, leading to reduced systemic exposure to substances administered via the IP route [40]. On the other hand, Li et al. have demonstrated that orally administered TA had minimal adverse side effects in rats [38]. However, the molecular composition of tannins in the bloodstream or excreted in urine may differ significantly from their ingested forms [41], and there is limited information available on the absorption, distribution, metabolism, and excretion (ADME) of hydrolyzable tannins in humans. This knowledge gap extends to trials conducted with rodents and pigs [42]. The aforementioned findings may raise concerns regarding the potential translation of these effects into clinical settings. Detailed discussions and exploration of potential mechanisms will be addressed in our future work.

In conclusion, our study demonstrates that the inclusion of TA significantly enhances cell viability and hepatic functions in both normal and APAP-injured liver models, both in vitro and in vivo, under hepatoprotective and hepatocurative conditions. Notably, these results highlight the pivotal role of TA, particularly its cumulative effect, in enhancing albumin synthesis in SHs over extended periods. Furthermore, TA’s potential for mitigating oxidative stress in hepatocytes highlights its promising applications in treating acute liver failure. This study offers scientific validation for the traditional belief that “drinking tea to protect the liver”. Specifically, the coadministration of TA-based components with APAP may hold potential for clinical applications.

### Limitations of the Study

In our study, we investigated the therapeutic potential of TA for APAP-induced liver injury through both in vitro and in vivo approaches. While our findings provide valuable insights, a more in-depth exploration of the underlying mechanisms, such as the immunoreactive levels of IL-1β and TNF-α and potential pathways, is warranted. Furthermore, it is important to note that although the IP route is commonly used in animal studies, its differences from oral intake, which is more relevant for human therapeutic application, should be considered. Future discussions will delve into the potential translation of these effects into clinical settings, with a focus on detailed mechanisms.

## 4. Materials and Methods

### 4.1. Chemicals, Animals, and Equipment

Rat Albumin ELISA Quantitation Set (E110-125) was purchased from Bethyl Laboratories (Montgomery, TX, USA). Urea Assay Kit (DIUR-100) was purchased from BioAssay Systems (Hayward, CA, USA). Propidium Iodide (40017) was purchased from Biotium (Fremont, CA, USA). DNA Quantity Kit (PMC-AK06-COS) was purchased from COSMO Bio (Tokyo, Japan). Annexin V, FITC Apoptosis Detection Kit (AD10) was purchased from Dojindo molecular technologies (Rockville, MD, USA). Trypan Blue Solution 0.4 *w*/*v*% (207-17081), collagenase (034-22363), tannic acid (201-06332), and glycerol (075-00616) were purchased from FUJIFILM Wako Pure Chemicals (Osaka, Japan). William’s E medium (CC901-0500) and DMEM/F12 medium (CC113-0500) were purchased from GeneDireX, Inc. (Taoyuan, Taiwan). qPCR primers (Albumin, GAPDH) were purchased from Genomics (New Taipei, Taiwan). Penicillin–Streptomycin (15140122), fetal bovine serum (26140079), and Insulin–Transferrin–Selenium (ITS-G) (100×) (41400045) were purchased from Gibco (Grand Island, NY, USA). MTT (3-(4,5-Dimethylthiazol-2-yl)-2,5-Diphenyltetrazolium Bromide) (M6494), TRIzol™ Reagent (15596026), and Cell Viability Imaging Kit, Blue/Green (R37609) were purchased from Invitrogen (Waltham, MA, USA). Nicotinamide (24317-72), 2-Propanol (29113-95), Albumin Bovine Serum (Fatty Acid Free) pH 7.0 (08587-42), Sodium chloride (31320-05), Potassium chloride (28514-75), tri-Sodium Phosphate 12-water (31804-75), di-Sodium Hydrogenphosphate 12-water (31723-35), Sodium Dodecyl Sulfate (31607-65), Sodium hydroxide (31511-05), Phenol red (26807-21), Tris (hydroxymethyl) aminomethane (35406-75), Sodium Carbonate (31310-35), Sodium Tetraborate Decahydrate (31223-85) were purchased from Nacalai (Kyoto, Japan). Isoflurane was purchased from Panion and BF Biotech (Taipei, Taiwan). Rotor-Gene SYBR Green PCR Kit (204001) and Rotor-Gene Q (9001580) were purchased from QIAGEN (Hilden, Germany). Lactate dehydrogenase activity assay kit (MAK066) glutaraldehyde solution (50% in H_2_O) (340885), Chloroform (288306), carbon tetrachloride (289116), Dimethyl sulfoxide (D4540), D-(+)-Galactosamine hydrochloride (G0500), Hydrochloric acid (320331), Urethane (U2500), L-Ascorbic acid 2-phosphate sesquimagnesium salt hydrate (A8960), L-Proline (P0380), Dexamethasone (D4902), PeroxiDetect™ Kit (PD1), Dimethyl sulfoxide (D5450), Glycine (410225), Ammonium sulfate (A4915), Methanol (179337), 2-Mercaptoethanol (M3148), HEPES (H3375), RNase A, Protease-Free (556746), Mayer’s Hematoxylin Solution (MHS32) were purchased from Sigma-Aldrich (Munich, Germany). The 1st strand cDNA Synthesis Kit (6110A) was purchased from TaKaRa (Shiga, Japan). UltraPure DNase/RNase-Free Distilled water (10977015) was purchased from Thermo Fisher Scientific (Waltham, MA, USA). A light microscope (Axio Vert. A1) was procured from Carl Zeiss AG (Oberkochen, Germany). A UV/Vis spectrophotometer (V-530) was purchased from JASCO (Tokyo, Japan). A microplate absorbance reader (Sunrise) was purchased from Tecan (Männedorf, Switzerland). A TurboCycler lite PCR thermal cycler (TCLT-9610) was purchased from Blue-Ray Biotech (Taipei, Taiwan). Rotor-Gene Q was purchased from QIAGEN (Venlo, The Netherlands).

### 4.2. Hepatocyte Isolation and Seeding

Hepatocytes (both MHs and SHs) were obtained from male SD rats (age, 6–8 weeks) by using a two-step collagenase perfusion technique with some modifications [13,43]. The Trypan Blue exclusion method indicated that the viability of each experimental preparation exceeded 85%. In total, 5 × 10^5^ cells/mL of hepatocytes (500 μL/well) were seeded on a 0.1 g/L collagen-coated 24-well plate. MHs were maintained in William’s E (WE) medium supplemented with 10% fetal bovine serum (FBS), whereas SHs were maintained in a DMEM/F12-based medium in both cultures. The medium was replaced with a 1 μg/mL TA-containing medium at 4 h and 24 h post-inoculation and subsequently changed every 48 h. Hepatocyte morphology was investigated using a microscope.

### 4.3. Injured MHs Cultures

MHs were cultured in WE medium containing 10% FBS, and the medium was replaced after 4 h. Then, the culture medium was replaced with WE medium + 1 μg/mL TA + 10% FBS and incubated for 20 h to protect MHs. Subsequently, the culture medium was replaced with WE medium + 20 mM APAP + 10% FBS and incubated for 18 h to induce injury in MHs. Furthermore, the culture medium was replaced again with WE medium + 1 μg/mL TA+ 10% FBS and incubated for another 18 h to facilitate the recovery of MHs from injury.

MHs refer to normal MHs cultured in regular Petri dishes without TA supplementation. APAP-MHs refer to APAP-injured MHs cultured in regular Petri dishes without TA supplementation. [TA + (APAP-MHs)] refers to MHs cultured in regular Petri dishes with TA supplementation, followed by exposure to APAP-induced toxicity (prevention model). [TA + (APAP-MHs) + TA] refers to MHs cultured in regular Petri dishes with TA supplementation, followed by exposure to APAP-induced toxicity for 18 h, and then recovered through additional TA supplementation for another 18 h (curative model).

### 4.4. Injured SHs Cultures

SHs were cultured in DMEM/F-12-based medium, and the medium was replaced after 4 h. Subsequently, the culture medium was replaced with DMEM/F-12-based medium + 1 μg/mL TA and incubated for 7 days to protect SHs. Then, the culture medium was replaced with DMEM/F-12-based medium + 20 mM APAP and incubated for 48 h to induce injury in SHs. Furthermore, the culture medium was replaced again with DMEM/F-12 medium + 1 μg/mL TA and incubated for another 24 h to facilitate the recovery of SHs from injury.

SHs refer to normal SHs cultured in regular Petri dishes without TA supplementation. APAP-SHs refer to APAP-injured SHs cultured in regular Petri dishes without TA supplementation. [TA + (APAP-SHs)] refers to SHs cultured in regular Petri dishes with TA supplementation, followed by exposure to APAP-induced toxicity (prevention model). [TA + (APAP-SHs) + TA] refers to SHs cultured in regular Petri dishes with TA supplementation, followed by exposure to APAP-induced toxicity for 48 h, and then recovered through additional TA supplementation for another 24 h (curative model).

### 4.5. Relative Hepatocyte Viability

The relative hepatocyte viability of normal and injured hepatocytes was examined using an MTT assay [44,45]. MTT was added to a final concentration of 10% in WE medium and incubated at 37 °C under 5% CO_2_ in a humidified incubator. After 4 h of incubation, 75 µL of the supernatant was aspirated, and 55 µL of dimethyl sulfoxide was added to each well. After another 10 min of incubation, absorbance was measured at 570 nm on a microplate reader. To calculate relative hepatocyte viability within the 5 days of culture, the measured absorbance value of hepatocytes in the medium without TA on day 1 was used as the blank. Subsequently, the absorbance value of each experimental condition was divided by the value of this blank to determine relative hepatocyte viability [44].

### 4.6. Assessment of Hepatic Functions

The culture medium collected in the previous step was stored at −20 °C for subsequent liver function analyses, specifically focusing on two key indicators: albumin synthesis and urea production [13,46]. The concentrations of albumin and urea in the collected media were determined using a protein detector ELISA kit, a horseradish peroxidase/ABTS system, and a QuantiChrom urea assay kit, respectively, following the manufacturer’s instructions.

### 4.7. Total RNA Extraction and Reverse Transcription

Total RNA was extracted from the cultured hepatocytes by using TRIzol [47]. The quality of the extracted RNA was assessed by determining the OD260/OD280 absorption ratio. Subsequently, total RNA (500 ng) was reverse-transcribed into single-stranded cDNA by using PrimeScript RTase and 50 pM random hexamer primers. Polymerase chain reaction (PCR) was performed using primers specific for liver function markers (albumin) and a housekeeping gene (glyceraldehyde-3-phosphate dehydrogenase [GAPDH]). The following primers were used: 96 bp albumin (forward, 5′-TCCCAGACAAGGAGAAGCAG-3′; reverse, 5′-TCACCGTCTTCAGCTGATCTT-3′) and 143 bp GAPDH (forward, 5′-GGCACAGTCAAGGCTGAGAATG-3′; reverse, 5′-ATGGTGGTGAAGACGCCAGTA-3′).

### 4.8. Real-Time PCR

In the rotor-gene SYBR Green PCR method, a mixture was prepared by combining 8 μL of RNAse-free water (PCR grade), 0.5 μL of forward primer (20 μM), 0.5 μL of reverse primer (20 μM), and 10 μL of rotor-gene SYBR Green master mix. Then, cDNA (1 μL) was amplified using a rotor-gene SYBR Green PCR kit and a standardized PCR protocol. The melting curve analysis program of the Rotor-Gene Q was used to identify specific PCR products. Gene expression data obtained from the analysis were quantified and normalized to the expression data of GAPDH. Relative gene expression was quantified in terms of fold change.

### 4.9. Relative Oxidative Stress

Disrupted liver metabolism is closely associated with inflammation and excessive ROS production, thereby promoting hepatocyte damage, and leading to liver diseases [23]. To assess the markers of lipid peroxidation, the PeroxiDetect kit was utilized in accordance with the manufacturer’s instructions. Specifically, 50 to 100 μL of the culture medium was added to a 1.5 mL Eppendorf tube, followed by the addition of 1 mL of the working color reagent. The mixture was left at room temperature for 30 min. Absorbance was measured at 560 nm by using a spectrophotometer. To calculate relative oxidative stress, the absorbance of the APAP-MHs cultured in the TA-free culture medium was used as the blank. Then, the absorbance value derived from each experimental condition was divided by the value of the blank to estimate relative oxidative stress.

### 4.10. Annexin V Detection Using a Flow Cytometer

Annexin V is a calcium-dependent phospholipid-binding protein that specifically binds to phosphatidylserine. Apoptosis can be detected using flow cytometry or fluorescence microscopy with green fluorescent fluorescein isothiocyanate (FITC)-labeled Annexin V [48]. Annexin V/propidium iodide (PI) staining and analysis were performed following the manufacturer’s instructions. Initially, as the cell culture progressed to day 1, cells on the culture plate were detached through trypsinization and suspended in phosphate-buffered saline. After centrifugation, the cells were resuspended in a solution conducive to Annexin V binding, resulting in a final cell concentration of 1 × 10^6^ cells/mL. Subsequently, 100 μL of this cell suspension was transferred to a new centrifuge tube, and 5 μL of FITC-labeled Annexin V and 5 μL of PI were added. The reaction occurred at room temperature for 15 min. Finally, the stained cells were analyzed through flow cytometry.

### 4.11. In Vivo Acute Liver Failure Therapeutic Model Establishment

C57Bl/6 mice, aged 7 weeks, were initially subjected to an 8 h fasting period, followed by an intraperitoneal injection of APAP. APAP was dissolved in 0.9% saline at 37 °C, and the injection dose was 300 mg/kg. Acute liver failure was induced within 12 h of poisoning, as described in [49]. Subsequently, we administered 300 μL of 1 μg/mL TA through an intraperitoneal injection to initiate a 12 h recovery period for APAP-induced liver failure. Experimental parameters were adapted from [24].

### 4.12. Biochemical Analysis

Blood samples were collected from the mice through cardiac puncture by using a 27 G 1 mL syringe. The samples were then centrifuged at 3000 rpm at 4 °C for 25 min. The resulting supernatants were carefully collected into 1.5 mL heparin-free microcentrifuge tubes and stored at −20 °C. The serum was maintained at 2 °C to 8 °C and sent to the Laboratory Animal Center, National Taiwan University College of Medicine, for further analysis of ALT, AST, and BIL-T. ALT and AST, specific enzymes of hepatocytes, are released into the bloodstream during hepatocytic necrosis, whereas BIL-T serves as an indicator of acute liver failure, as described in [50].

### 4.13. Histological Analysis

After the 12 h in vivo TA injection, livers were extracted and fixed in formalin, embedded in paraffin, and sliced into sections for histological staining. The paraffin-embedded sections were stained using hematoxylin and eosin (H and E). H and E staining, which uses hematoxylin to stain cell nuclei blue–purple and eosin to stain the cytoplasm pink, was conducted to observe and analyze cellular morphology [46,51]. Paraffin embedding and staining procedures were conducted in the Sectioning Room of the Department of Veterinary Medicine at National Taiwan University.

### 4.14. Statistical Analysis

All data are presented as the mean ± standard deviation. Each experiment was conducted in biological triplicate. Statistical analyses involving multiple samples were conducted using a two-tailed unpaired Student’s *t* test using Microsoft Excel 2017. *p* < 0.05 indicated statistical significance.

## 5. Conclusions

In our study, we observed a significant enhancement in the relative viability and hepatic functions of both MHs and SHs in vitro at a specific concentration of TA. Furthermore, our findings highlight the hepatoprotective and hepatocurative effects of TA against APAP-induced hepatotoxicity. Additionally, our results underscore the therapeutic potential of TA through IP administration in clinical settings. Future investigations should comprehensively explore the mechanisms underlying TA’s protective and preventive effects on hepatocytes, whether administered orally, and assess its long-term impact on liver function in cases of liver injury.

## Data Availability

The data presented in this study are available on request from the corresponding author. The data are not publicly available due to privacy or ethical restrictions.
